# Maternal age and child morbidity: A Danish national cohort study

**DOI:** 10.1371/journal.pone.0174770

**Published:** 2017-04-05

**Authors:** Malene Meisner Hviid, Charlotte Wessel Skovlund, Lina Steinrud Mørch, Øjvind Lidegaard

**Affiliations:** 1 Department of Gynecology, Zealand University Hospital, Roskilde, Denmark; 2 Department of Gynecology, Rigshospitalet, University of Copenhagen, Copenhagen, Denmark; University of Bremen, GERMANY

## Abstract

**Introduction:**

The mean age at delivery has increased over the latest half of a century. Women of advanced maternal age have increased obstetrical risks and increased risk of chromosomal abnormalities and some other specified diagnoses in the offspring. The aim of this study was to assess the association between maternal age and overall child morbidity according to main diagnosis groups.

**Material and methods:**

We conducted a national cohort study including 352 027 live firstborn singleton children. The children were born between Jan 1994 and Dec 2009 and followed to Dec 2012. Children were divided into groups according to maternal age: 15–24, 25–29, 30–34, and 35+ years. Poisson regression analyses calculated adjusted incidence rate ratios (IRR) of child morbidities according to main diagnoses groups A-Q of the International Classification of Disease 10 with adjustment for year of birth, body mass index, smoking, and mother’s level of education.

**Results:**

Average follow-up time was 11 years. Compared to children born to women 25–29 years, firstborn children to mothers aged 35+ had higher child morbidity in 8 of 19 main diagnosis groups and firstborn children to mothers 15–24 years had higher child morbidity in 12 of 19 main diagnosis groups. Thus, for a majority of diseases a U-shaped correlation was found, with lowest rates in women 25–29 years.

**Conclusion:**

Firstborn children to both older and younger mothers have higher overall morbidity as compared to children born by mothers 25–29 years.

## Introduction

The average age at first delivery has in Denmark increased from 22.7 years in 1965 to 29.1 years in 2013 [1]. Among suggested reasons for postponing parenthood are the ability to prevent and plan pregnancy with simple and effective contraceptive methods, problems by finding a suitable partner, women pursuing education and career before parenthood, and increased prosperity [2].

Advanced maternal age has been associated with several adverse maternal and perinatal outcomes, such as reduced fertility, increase in risk of miscarriages, preterm birth, large for gestational age, stillbirth, and to increase the rate of Caesarean sections and instrumental delivery [3–6]. Furthermore, advanced maternal age increases the risk of gestational diabetes, hypertension, and preeclampsia [7–12]. It has been argued that these age-related diseases are mediators of perinatal pregnancy outcomes such as low birth weight, children born small for gestational age, and preterm delivery [7,12].

Young maternal age has been associated with a significantly lower risk of preeclampsia, post-partum haemorrhage, instrumental delivery, a decreased rate of Caesarean section, an increased risk of preterm birth, and a significantly increased risk of fetal death [13–15].

Adverse pregnancy exposures are believed to affect long-term health in the offspring [16,17]. Finally, it is known that children born by older mothers have an increased risk of chromosomal abnormalities, such as Downs Syndrome [18]. Few studies have assessed long-term health in children born by mothers of different ages [19,20]. A variety of studies have linked specific diagnosis in children to advanced maternal age, such as type 1 diabetes, childhood cancers, and childhood autism [21–26]. We found no study investigating the potential impact of advanced maternal age or young maternal age on general long-term child morbidity.

Therefore, the aim of this study was to assess the association between maternal age and overall child morbidity for each of the main diagnosis groups A-Q within the ICD10 among all live born in Denmark. We conducted our analyses on firstborn singleton children.

## Materials and methods

We conducted a National cohort study including all live born singletons in Denmark from 1^st^ of January 1994 through December 31^st^, 2009 and followed the children till December 31, 2012. The children were grouped according to maternal age at childbirth: 15–24, 25–29, 30–34, and 35+ years. Overall child morbidity was measured counting ICD-10 diagnosis codes and was categorized according to the first letter in the diagnosis codes A-Q, each letter categorising a specific area of disease, for example F covering all mental disorders. We counted both primary and secondary diagnoses among the children. Diagnoses with a typical short disease period were counted several times, after a restriction period of 12 weeks. We considered bacterial infections, non-bacterial infections, benign tumors, anemia, and ear diseases as such diagnoses. All other diagnosis groups were defined as chronic diseases and only counted once at the time of first diagnosis. Since diagnosis only counted once or after a restriction period of 12 weeks, transfers between hospitals were thus taken into account.

We defined mothers at 35 years or older as mothers of advanced age. Smoking was categorized as either current smoker or non-current smoker. Body mass index was categorised according to World Health Organisation; <18.5 Underweight, 18.5–24.9, normal range (reference) ≥25–30, overweigh ≥30 Obese. Smoking and BMI were registered in first trimester of pregnancy. Educational length had four levels: a) unknown or no education, b) primary education or lower secondary education, c) upper secondary education or post secondary non-tertiary education, and d) first- or second stage of tertiary education.

### Data and registers

We collected data from two National registers; The National Health Register holds by law information on discharge diagnoses and hospital stay from all public and private hospitals, including information on antenatal care, childbirth, and the post-partum period. The register contains all discharge diagnosis since 1977 and since 1995 also all outpatient diagnoses, main as well as secondary diagnosis. The National Health Register is maintained by the Health Data Board and all information registered is delivered continuously throughout the country. The register is used to measure hospital activity, monitor diseases and treatments, and to aid in medical research. The National Health Register delivered information on maternal age, maternal body mass index, and smoking at the time of pregnancy, and for the children; birth weight, gestational age, and ICD-10 diagnosis codes. Status on emigration and maternal education was delivered from Statistics Denmark. Statistics Denmark works as an independent unit under the Danish government, providing official accessible statistics. Statistics are used in politics and research. Data from the registries were linked by unique identification numbers given to all Danish citizens at birth or when accepted citizenship is achieved by immigrants.

### Statistical analyses

Children were followed from birth until time of emigration, time of death, or December 31^st^, 2012. However, those who moved back to Denmark contributed with person-time also while emigrated. Relative risks of child morbidities were calculated by Poisson regression using SAS 9.4 with children born by mothers 25–29 years as reference. Children’s age was used as risk time to account for differences in follow-up time.

The analyses were adjusted for possible confounding influence from year of birth, maternal body mass index, smoking, and educational length. Being born preterm and small for gestational age were considered a consequence of maternal age (mediators) and therefore not adjusted for. As parity is known to influence child morbidity, firstborns being associated with increased morbidity, our main results were made on only firstborn children. Stratified analyses were made to assess effect modification from maternal education on the association between maternal age and child morbidity. The study was approved by the Danish Data Protection Agency, journal number 2012-41-0605 and approved by Statens Serum Institute. Since the study only uses data already registered for clinical use, no further ethical approval was necessary.

## Results

During the 16-year study period, 1 017 577 singletons were born in Denmark. After exclusion of stillborn, 1 014 280 children remained, including 99.7% of all newborn singletons in Denmark. Of the 1 014 280 delivered children, 352 027 were firstborn (Table 1). The children were followed from delivery and three to 19 years, on average 11 years.

**Table 1:** Characteristics of delivering women in different age groups.

Maternal age group/years	15–24	25–29	30–34	35+	All ages
Firstborn children (n)	78 503	150 598	93 236	29 690	352 027
All children (n)	147 339	359 709	349 729	157 503	1 014 280
Firstborn (%)	53.3	41.9	26.7	18.9	34.7
Born before 37 weeks (%)	5.5	4.7	4.4	5.3	4.8
Birth weight <2500 g (%)	4.7	3.5	3.4	4.4	3.8
Smoking (%)	20.3	11.9	10.6	12.5	12.8
BMI>25 (%)	9.4	10.5	12.1	14.1	11.5
Education level 0^a^ (%)	3.0	1.8	1.6	2.4	2.0
Education level 1^b^ (%)	44.5	16.9	12.3	14.3	18.9
Education level 2^c^ (%)	41.0	44.5	38.8	34.5	40.5
Education level 3^d^ (%)	9.5	35.6	46.5	47.9	37.5

^a^ Educational level 0 = Unknown or no education

^b^ Educational level 1 = primary education or lower secondary education

^c^ Educational level 2 = upper secondary education or post secondary non-tertiary education

^d^ Educational level 3 = first- or second stage of tertiary education

### Mothers 35+ years

Firstborn of mothers of advanced age had a statistically significantly increased morbidity in eight of 19 main diagnosis groups including mental disorders, cerebral-, eye-, heart-, circulatory-, rheumatic-, neonatal diseases, and congenital malformations (Table 2). Incidence rate ratios (RR) ranged from 1.06 to 1.29. Decreased morbidity was found for airway diseases; RR 0.97 (0.94–1.00). There was as compared with the reference population of children born by mothers 25–29 years no difference in morbidity in children born to mothers of advanced age for the majority of main diagnosis groups including; bacterial infections, non-bacterial infections, malignant diseases, benign tumors, anemia, endocrine disorders, ear diseases, gastro-intestinal diseases, dermatological disorders, and kidney, genital- and urinary tract diseases.

**Table 2:** Morbidity in firstborn singletons according to maternal age.

Diagnosis groups	Age	Events	No. of children	Crude RR	Adjusted RR^b^	95% confidens interval		P-value
**Bacterial infections**^a^	15–24	8767	78 503	**1.41**	**1.25**	1.21	1.30	<0.001
	25–29	12 021	150 598	1.00	1.00	Reference		
	30–34	7027	93 236	**0.95**	**0.97**	0.94	1.00	0.04
	35–49	2350	29 690	1.01	1.01	0.96	1.06	0.67
**Non-bacterial infections**^a^	15–24	8035	78 503	**1.33**	**1.20**	1.16	1.24	<0.001
	25–29	11 609	150 598	1.00	1.00	Reference		
	30–34	7098	93 236	1.00	1.03	1.00	1.06	0.06
	35–49	2282	29 690	1.02	1.02	0.98	1.07	0.34
**Malignant diseases**	15–24	105	78 503	0.87	0.82	0.62	1.08	0.16
	25–29	224	150 598	1.00	1.00	Reference		
	30–34	138	93 236	1.06	0.96	0.76	1.22	0.74
	35–49	53	29 690	1.32	1.21	0.86	1.69	0.27
**Benign tumors**^a^	15–24	1594	78 503	0.97	1.05	0.98	1.13	0.17
	25–29	3122	150 598	1.00	1.00	Reference		
	30–34	1916	93 236	1.03	1.02	0.96	1.09	0.51
	35–49	591	29 690	1.02	1.02	0.93	1.13	0.64
**Anemia**^a^	15–24	1698	78 503	**1.26**	**1.19**	1.10	1.28	<0.001
	25–29	2559	150 598	1.00	1.00	Reference		
	30–34	1448	93 236	0.94	0.96	0.89	1.03	0.24
	35–49	438	29 690	0.92	0.96	0.86	1.07	0.41
**Endocrine disorders**	15–24	3747	78 503	**1.24**	**1.14**	1.08	1.19	<0.001
	25–29	5801	150 598	1.00	1.00	Reference		
	30–34	3296	93 236	**0.93**	**0.94**	0.90	0.99	0.01
	35–49	1111	29 690	1.00	0.99	0.93	1.07	0.86
**Mental disorders**	15–24	2372	78 503	**1.39**	**1.12**	1.05	1.20	<0.001
	25–29	3146	150 598	1.00	1.00	Reference		
	30–34	1783	93 236	0.98	0.99	0.93	1.05	0.71
	35–49	641	29 690	**1.15**	**1.15**	1.05	1.26	0.003
**Cerebral diseases**	15–24	2428	78 503	**1.18**	**1.07**	1.00	1.14	0.04
	25–29	3859	150 598	1.00	1.00	Reference		
	30–34	2331	93 236	1.03	1.04	0.98	1.10	0.22
	35–49	800	29 690	**1.14**	**1.12**	1.03	1.22	0.01
**Eye diseases**	15–24	3125	78 503	**1.16**	**1.08**	1.03	1.14	0.004
	25–29	5064	150 598	1.00	1.00	Reference		
	30–34	3144	93 236	**1.05**	1.05	1.00	1.10	0.06
	35–49	1145	29 690	**1.24**	**1.21**	1.13	1.30	<0.001
**Ear diseases**^a^	15–24	9056	78 503	**1.23**	**1.11**	1.08	1.15	<0.001
	25–29	13 909	150 598	1.00	1.00	Reference		
	30–34	8156	93 236	0.98	1.00	0.97	1.03	0.96
	35–49	2676	29 690	1.04	1.03	0.99	1.08	0.17

Relative risk; RR, Mothers ages 25–29 were used as reference indicated by RR 1.00

^a^ Diagnosis reoccurring through life, counted again after a restriction period of 12 weeks.

^b^ Adjusted for calendar year, smoking status, BMI, and maternal educational level.

### Mothers 15–24 years

Among firstborn children to young mothers (aged 15–24 years) we found higher morbidity in 12 of 19 main diagnosis groups, as compared to children to mothers 25–29 years old (Tables 2 and 3), with RR ranging from 1.06 to 1.25. The 12 main diagnosis groups included bacterial infections, non-bacterial infections, anemia, endocrine diseases, mental disorders, cerebral-, eye-, ear-, airway-, gastro-intestinal diseases, dermatological disorders, and kidney, genital- and urinary tract diseases. Rates of neonatal diseases were lower among children born to young mothers. There was no difference in morbidity in six of the 19 main diagnosis groups including malignant diseases, benign tumors, heart-, circulatory-, rheumatic diseases, and congenital malformations.

**Table 3:** Morbidity in firstborn singletons according to maternal age.

Diagnosis groups	Age	Events	No. of children	Crude RR	Adjusted RR^a^	95% confidens interval		P-value
**Heart diseases**	15–24	367	78 503	**1.22**	1.06	0.90	1.24	0.50
	25–29	568	150 598	1.00	1.00	Reference		
	30–34	391	93 236	**1.15**	**1.16**	1.01	1.34	0.04
	35–49	129	29 690	1.21	**1.29**	1.05	1.58	0.02
**Circulatory diseases**	15–24	435	78 503	1.01	1.00	0.87	1.15	0.10
	25–29	817	150 598	1.00	1.00	Reference		
	30–34	458	93 236	0.95	0.96	0.85	1.09	0.56
	35–49	177	29 690	1.17	**1.21**	1.01	1.45	0.04
**Airway diseases**	15–24	20 991	78 503	**1.25**	**1.15**	1.13	1.18	<0.001
	25–29	32 259	150 598	1.00	1.00	Reference		
	30–34	18 640	93 236	**0.95**	**0.96**	0.94	0.98	<0.001
	35–49	5974	29 690	0.97	**0.97**	0.94	1.00	0.03
**Gastro-intestinal diseases**	15–24	8395	78 503	**1.20**	**1.09**	1.05	1.12	<0.001
	25–29	13 275	150 598	1.00	1.00	Reference		
	30–34	7856	93 236	0.99	0.99	0.96	1.02	0.67
	35–49	2532	29 690	1.02	1.02	0.97	1.07	0.42
**Dermato-logical disorders**	15–24	4881	78 503	**1.20**	**1.13**	1.08	1.17	<0.001
	25–29	7712	150 598	1.00	1.00	Reference		
	30–34	4649	93 236	1.01	1.01	0.97	1.05	0.61
	35–49	1497	29 690	1.05	1.05	0.99	1.12	0.11
**Rheumatic diseases**	15–24	6221	78 503	1.02	1.01	0.97	1.05	0.74
	25–29	11 348	150 598	1.00	1.00	Reference		
	30–34	6684	93 236	1.01	1.00	0.97	1.04	0.84
	35–49	2143	29 690	**1.05**	**1.06**	1.01	1.12	0.02
**Kidney and urinary diseases**	15–24	4608	78 503	**1.06**	**1.06**	1.02	1.11	0.01
	25–29	8155	150 598	1.00	1.00	Reference		
	30–34	4681	93 236	0.97	0.97	0.93	1.01	0.17
	35–49	1457	29 690	0.98	1.00	0.94	1.06	0.93
**Neonatal diseases**	15–24	19 105	78 503	0.99	**0.94**	0.92	0.96	<0.001
	25–29	37 486	150 598	1.00	1.00	Reference		
	30–34	24 973	93 236	**1.06**	**1.07**	1.05	1.09	<0.001
	35–49	9047	29 690	**1.20**	**1.18**	1.15	1.21	<0.001
**Congenital malfor-mations**	15–24	7612	78 503	1.03	0.99	0.96	1.02	0.44
	25–29	14 243	150 598	1.00	1.00	Reference		
	30–34	9283	93 236	**1.07**	**1.08**	1.05	1.11	<0.001
	35–49	3152	29 690	**1.15**	**1.14**	1.10	1.19	<0.001

Relative risk; RR, Mothers ages 25–29 were used as reference indicated by RR 1.00

^**a**^ Adjusted for calendar year, smoking status, BMI, and maternal educational level.

### Association between maternal educational level and child morbidity

We found a consistent increasing morbidity with decreasing maternal educational level for eight of 19 main diagnoses groups including bacterial infections, non-bacterial infections, anemia, endocrine disorders, heart diseases, dermatological disorders, gastro-intestinal diseases, and neonatal diseases. Benign tumor’s on the other hand increased with increasing educational length. Further six diagnosis groups showed a non-consistent but nevertheless increased morbidity with shorter education including mental disorders, cerebral diseases, eye diseases, ear diseases, airway diseases, and congenital malformations (Tables 4 and 5). The influence of maternal age was generally enhanced in strata with long education as compared with the influence of age in women with shorter education. In the strata with unknown or no education, child morbidity was not associated to maternal age.

**Table 4:** Child morbidity according to maternal educational level.

Diagnosis groups	Age	Education level 0^a^	Education level 1^b^	Education level 2^c^	Education level 3^d^
**Bacterial infections**	**All**	**1.54**	**1.40**	**1.18**	**1.00**
	15–24	0.92	**1.18**	**1.31**	**1.30**
	25–29	1.00	**1.00**	**1.00**	1.00
	30–34	0.92	**0.90**	**0.92**	**0.92**
	35–49	0.96	**0.85**	**0.90**	**0.92**
**Non-bacterial infections**	**All**	**1.46**	**1.30**	**1.06**	**1.00**
	15–24	0.90	**1.12**	**1.20**	**1.17**
	25–29	1.00	**1.00**	**1.00**	1.00
	30–34	**0.86**	**0.92**	**0.94**	1.02
	35–49	0.87	**0.88**	**0.94**	0.99
**Malignant diseases**	**All**	0.71	0.98	1.00	1.00
	15–24	1.39	0.82	0.84	0.91
	25–29	1.00	1.00	1.00	1.00
	30–34	1.20	0.91	1.03	**0.79**
	35–49	1.14	1.02	1.23	0.88
**Benign tumors**	**All**	**0.67**	**0.88**	**0.91**	**1.00**
	15–24	0.88	1.11	1.05	**1.21**
	25–29	1.00	1.00	1.00	1.00
	30–34	0.87	1.04	**0.93**	0.99
	35–49	0.73	0.94	1.02	**0.88**
**Anemia**	**All**	**1.43**	**1.27**	**1.12**	**1.00**
	15–24	**0.67**	**1.14**	**1.30**	**1.24**
	25–29	1.00	**1.00**	1.00	1.00
	30–34	0.80	1.00	1.01	**0.88**
	35–49	**1.63**	1.11	1.00	**0.85**
**Endocrine disorders**	**All**	**1.51**	**1.35**	**1.17**	**1.00**
	15–24	1.00	**1.09**	**1.20**	**1.13**
	25–29	1.00	**1.00**	1.00	1.00
	30–34	0.93	0.96	**0.90**	**0.89**
	35–49	1.09	**0.87**	**0.91**	**0.93**
**Mental disorders**	**All**	**1.24**	**1.64**	**1.19**	**1.00**
	15–24	0.72	**1.18**	**1.15**	**1.20**
	25–29	1.00	**1.00**	1.00	1.00
	30–34	0.89	0.96	**0.86**	**0.91**
	35–49	0.94	0.97	0.93	**0.90**
**Cerebral diseases**	**All**	**1.27**	**1.29**	**1.11**	**1.00**
	15–24	0.99	1.03	**1.14**	1.08
	25–29	1.00	1.00	1.00	1.00
	30–34	1.02	0.95	1.01	0.99
	35–49	0.84	0.97	1.06	0.97
**Eye diseases**	**All**	**1.16**	**1.22**	**1.05**	**1.00**
	15–24	1.01	1.07	**1.16**	1.06
	25–29	1.00	1.00	1.00	1.00
	30–34	1.01	1.00	0.95	0.97
	35–49	0.91	1.02	0.99	1.00

Educational level 3 used as reference. Adjusted for BMI, smoking, and age.

^a^ Educational level 0 = Unknown or no education,

^b^ Educational level 1 = primary education or lower secondary education,

^c^ Educational level 2 = upper secondary education or post secondary non-tertiary education,

^d^ Educational level 3 = first- or second stage of tertiary education

**Table 5:** Child morbidity according to maternal educational level.

Diagnosis groups	Age	Education level 0^a^	Education level 1^b^	Education level 2^c^	Education level 3^d^
**Ear diseases**	**All**	**1.27**	**1.29**	**1.12**	**1.00**
	15–24	0.93	**1.05**	**1.15**	1.00
	25–29	1.00	**1.00**	1.00	1.00
	30–34	0.91	**0.92**	**0.97**	**0.97**
	35–49	0.87	**0.89**	**0.90**	**0.93**
**Heart diseases**	**All**	**1.57**	**1.43**	**1.11**	**1.00**
	15–24	0.58	1.07	1.13	1.00
	25–29	1.00	1.00	1.00	1.00
	30–34	0.68	1.18	1.04	0.99
	35–49	0.75	0.97	**1.25**	0.94
**Circulatory diseases**	**All**	1.19	1.00	1.00	1.00
	15–24	0.67	1.08	1.15	0.91
	25–29	1.00	1.00	1.00	1.00
	30–34	0.55	0.97	0.91	0.92
	35–49	0.70	0.92	0.96	0.93
**Airway diseases**	**All**	**1.19**	**1.22**	**1.07**	**1.00**
	15–24	1.09	**1.08**	**1.12**	**1.10**
	25–29	1.00	**1.00**	1.00	1.00
	30–34	0.94	**0.93**	**0.92**	**0.98**
	35–49	1.00	**0.88**	**0.90**	**0.95**
**Gastro- intestinal diseases**	**All**	**1.23**	**1.23**	**1.10**	**1.00**
	15–24	0.99	**1.11**	**1.12**	**1.11**
	25–29	1.00	**1.00**	1.00	1.00
	30–34	0.97	**0.95**	**0.92**	**0.93**
	35–49	0.97	0.98	**0.90**	**0.89**
**Dermatological disorders**	**All**	**1.44**	**1.23**	**1.03**	**1.00**
	15–24	1.02	**1.14**	**1.12**	**1.11**
	25–29	1.00	**1.00**	1.00	1.00
	30–34	**0.83**	0.96	**0.95**	1.00
	35–49	0.91	**0.92**	**0.92**	1.01
**Rheumatic diseases**	**All**	**0.92**	0.99	**1.02**	1.00
	15–24	0.96	1.00	1.03	1.04
	25–29	1.00	1.00	1.00	1.00
	30–34	1.06	**0.91**	**0.96**	**0.96**
	35–49	1.06	0.95	**0.92**	**0.94**
**Kidney and urinary diseases**	**All**	1.04	1.01	0.98	1.00
	15–24	0.89	1.01	**1.18**	**1.13**
	25–29	1.00	1.00	1.00	1.00
	30–34	0.86	**0.92**	**0.92**	**0.90**
	35–49	0.95	**0.91**	**0.93**	**0.89**
**Neonatal diseases**	**All**	**1.19**	**1.10**	**1.05**	**1.00**
	15–24	**0.90**	1.01	**1.06**	**1.07**
	25–29	1.00	1.00	1.00	1.00
	30–34	0.99	**1.05**	0.99	**0.94**
	35–49	1.07	**1.18**	**1.08**	0.98
**Congenital malformations**	**All**	**1.09**	**1.11**	**1.05**	**1.00**
	15–24	0.90	1.04	**1.05**	1.04
	25–29	1.00	1.00	1.00	1.00
	30–34	1.15	0.95	0.99	1.00
	35–49	1.01	**1.08**	1.00	0.98

Educational level 3 used as reference. Adjusted for BMI, smoking, and age.

^a^ Educational level 0 = Unknown or no education,

^b^ Educational level 1 = primary education or lower secondary education,

^c^ Educational level 2 = upper secondary education or post secondary non-tertiary education,

^d^ Educational level 3 = first- or second stage of tertiary education

## Discussion

Despite the decreased fertility and increased obstetrical risks women face getting pregnant at advanced age, maternal age continues to increase in industrialized countries. Besides increased risk of chromosomal malformations, low birth weight, small for gestational age, and preterm delivery among offspring of older mothers [7,12,27], recently also increased risks of other specific diseases have been suggested such as childhood cancers [23,24], type 1 diabetes [21,22] and childhood autism [25,26]. We found increased child morbidity among firstborn children to mothers aged 35+ in 8 of 19 main diagnosis groups and only a slightly decreased morbidity of airway diseases. Among young mothers, the literature suggests an increased risk of preterm delivery and low birth weight infants, and an increased risk of fetal death [13–15]. We found increased overall child morbidity in 12 of 19 main diagnosis groups among children to young mothers.

The spectrum of childhood diseases differed significantly between the young and the older mothers. Children to older mothers had increased morbidity in diagnosis groups generally not associated with infectious diseases for example mental disorders, heart diseases, circulatory diseases, and congenital malformations. Contrary, children to young mothers seem to have morbidities often associated with infectious diseases for example bacterial, nonbacterial infections, airway-, gastrointestinal-, and kidney, genital and urinary tract diseases. A possible mechanism explaining our findings may be the association between being born with low birth weight as a result of the intrauterine environment and diseases in later life [28]. The explanation may be that the cause of low-birth-weight among the children, differ between the two groups. Older mothers are known to suffer more from hypertension, preeclampsia, and gestational diabetes, all diseases associated with risk of low-birth weight infants [7,29,30]. However, the association between young age and increased risk of low birth weight infants remains unclear [13]. The higher incidence of infectious diseases in children to young mothers could be influenced by a lower threshold of referring their children to the health care sector in case of infections, as compared with older and perhaps more mature mothers. Furthermore, we were not able to adjust for an unhealthy lifestyle, like binge drinking and other unknown age-related lifestyle habits. Further studies are needed to evaluate this difference according to age in detail.

We found no previous study investigating the association between maternal age and overall child morbidity, most likely due to the lack of national databases that holds complete health information about all residents over several decades.

Myrskylä et al. investigated the association between maternal age and offspring adult health. Outcome was a frailty index, including eight different conditions. In addition also self-rated health, height, obesity, and mortality were assessed [19]. The study found that young maternal age <25 years and advanced maternal age >45 years were both associated with negative offspring health in adulthood, results in accordance with our findings.

Among mothers 35+ we found statistically significant although modestly increased morbidity in eight of 19 main diagnosis groups, including mental disorders. Several studies have investigated the association between maternal age and various mental disorders [25,26]. A Danish study by McGrath el al. assessed the association between maternal age and risk of any psychiatric disorder, and found a negative association to maternal age [31]. Thus, children born by mothers 12–19 years had a RR of any psychiatric disease of 1.51 (CI; 1.48–1.54), and children born by mothers 20–24 years a RR of 1.21 (CI; 1.19–1.21) as compared to children born by mothers 25–29 years old, whereas children to mothers aged 35+ had lower risk of any psychiatric disorder; RR 0.94 (CI; 0.92–0.96). In our study, we restricted the analysis to firstborn and found a 15% increased risk of mental disorders among children born by mothers 35+ and 12% increased risk among children to mothers 15–24 years. It thus appears that among firstborn children, maternal age 15–24 and 35+ implies higher risk for any psychiatric disorder. When we analysed the same association including all children, we found similar associations between maternal age and risk of mental disorder as found previously, suggesting a confounding influence by parity and plurality for this association in the McGrath study.

The consistent positive association between maternal age and neonatal diseases confirms previous studies demonstrating an increased risk of perinatal morbidity in children to older mothers such as intrauterine growth restriction, low birth weight, preterm birth, and neonatal mortality [5,7,10–12]. Joseph et al. found an association between advanced maternal age and perinatal morbidity assessing all children born after gestational age of 20 weeks and with a birth weight >500 grams, and including stillborn [29]. Blomberg et al. found an increased risk of small for gestational age, fetal distress, meconium aspiration, and Apgar <7 after 5 min among children to primiparous women over 30 years of age as compared to primiparous 25–29 years [32]. Our result on neonatal morbidity confirms previous findings of increased risk of perinatal morbidity among children of women aged 35+; RR 1.18 CI (1.15–1.21). We also found a significantly lower risk of neonatal diseases in newborn of young mothers. Further analysis of our results will determine which specific diagnosis code contributes to the shown increase and decrease in the old and young maternal age groups, respectively. Our results on neonatal morbidity including all children showed a weaker association to maternal age, suggesting parity to play a confounding influence on that association [3–5].

Mothers aged 30–34 years and 35+ had an increased risk of giving birth to a child with congenital malformation (both chromosomal and non-chromosomal). Our results match a study by Hollier et al. demonstrating an increased risk of both chromosomal and non-chromosomal malformations in children born by older mothers [27]. Another study by Baird et al. assessing only birth defects of unknown aetiology among liveborn, found a decreasing risk of two types of birth defects out of 43 tested with increasing maternal age and a bell-shaped association between hip-click and maternal age. The remaining 40 defects tested showed no statistically significant results [33]. A third population study by Croen et al. found a J-shaped association between maternal age and overall congenital malformations for all live born in California from 1983 through 1988. The lowest risk of malformations was found among children to mothers 25–29 years [34]. Of all the main diagnosis groups, congenital malformations had the strongest association to maternal age, which was almost unchanged with control for confounders.

We found no previous study investigating the association between maternal age and overall child morbidity of cerebral-, eye-, heart-, circulatory-, and rheumatic diseases, all of which were statistically significantly increased in children to mothers 35+. The 29% (5–58%) increased risk of heart diseases among children to older mothers should be confirmed and evaluated in future studies. Airway disease was the only diagnosis group with statistically significantly decreased morbidity in children to mothers aged 35+. Airway diseases include all respiratory tract infections, asthma, and allergies affecting the respiratory tract system.

In our study malignant diseases and benign tumours showed no association to maternal age. Several studies have investigated the association between maternal age and risk of childhood cancer. A large cohort study from Sweden, including 4.3 million children born from 1961 to 2000 found a positive association between advanced maternal age and retinoblastoma and leukaemia [23]. Another Swedish study by Hemminki et al. also found an increased risk of leukaemia among children born by older mothers [35]. A case-control study by Johnson et al. demonstrated a gradually increasing risk of childhood cancers with every five-year increase in maternal age [24]. Our results for childhood cancers demonstrated the same tendencies but not statistically significant, perhaps due to low number of children diagnosed with cancer, or because of diverse effects for specific types of cancer, since we examined all types of cancers combined into one outcome. A larger population study is needed to evaluate this association further.

Among mothers 15–24 years, we found increased morbidity in 12 of 19 main diagnosis groups. We found no study assessing the same association to maternal age, except to mental disorders as described above.

### Child morbidity and maternal educational level

In Denmark educational level is generally a better measure of socioeconomic status than income. Educations are government paid all the way through university, thereby in principle making higher education accessible for all citizens, regardless of social class.

Blackburn et al found an inverse association between socioeconomic status and child morbidity [36]. We found the same association between maternal educational level and child morbidity for a majority of the diagnosis groups. An exception was benign tumors, which increased with increasing educational level. We found no other study assessing this association. Interestingly, we found that children born by young mothers with no education or unknown education had significantly lower risk of anemia, compared to mothers aged 25–29 years. Risk of anemia increased with increasing maternal educational level. This is in contrast to an Indian study were low educational level and anemia among the mother significantly increases the risk of childhood anemia [37]. Our results are also in contrast to another study by de Vienne et al. who found an association between young age and risk of maternal anemia [13], which is known to increase the risk of anemia in the child. Reaching a plausible explanation to our findings is difficult without further investigation on the subject.

### Limitations and strengths

A large number of comparisons were carried out and some associations could therefore be due to chance. However, the consistency in our findings, support the overall conclusion of a small increased risk of child morbidity among children of older and young mothers.

We cannot exclude the possibility of spurious associations caused by potential unknown confounding factors, and studies within other populations should be carried out before causality can be established.

Our study examined associations to disease groups rather than to specific diseases, which could hide associations to specific sub-diagnoses. Opposite associations between maternal age and specific diseases would, when combined into one disease group, hide specific associations. It is likely that the observed associations are driven by the more common diagnoses within a diagnosis group. Future studies analysing a level deeper in the main diagnosis groups could clarify more specific associations. However, our main objective with this study was to assess the association between maternal age and overall child morbidity.

Validity differs between common diagnoses. That circumstance, however, will not affect the tested association to maternal age since registration validity of diagnoses should not be associated to maternal age. We were not able to adjust for differences in health behaviour among maternal age groups, and results should therefore be interpreted cautiously. Our study holds no information about the age of the father. The strong correlation between maternal and paternal age implies, that the demonstrated associations reflect a combined association to maternal age as well as paternal age. We were only able to adjust for a handful of possible confounders, other disease specific variables were not adjusted for and spurious findings among the results are possible.

Among the strengths were the generally high validity of discharge diagnoses from our hospitals [38], the complete capture of delivering women in Denmark, the relatively long follow-up period, and the almost complete follow-up rate, only limited by emigration.

It should be stressed, that our study aimed to assess the influence of maternal age by itself, and not the secondary offspring consequences due to age differences according parity, plurality and infertility, the two latter of which burden older delivering women, while parity burden younger women’s offspring.

In conclusion, firstborn children to both older (ages 35+) and younger mothers (aged 15–24) had slightly higher overall morbidity as compared to children born by mothers 25–29 years. We found an inverse association between level of maternal education and child morbidity in a majority of the main diagnosis groups. If these findings reflects biological mechanisms, they add to the list of consequences by early- as well as postponed parenthood, and it seems that the optimal maternal age for a child to be born considering child morbidity is between 25 and 34 years.

## Supporting information

S1 Table Summary of the main diagnosis groups and ICD-10 codes. (PDF)
