# Supplementary material for: Maternal age and child morbidity: A Danish national cohort study
Source: PLoS One. 2017 Apr 5;12(4):e0174770. doi: 10.1371/journal.pone.0174770 (PMC5381873; doi:10.1371/journal.pone.0174770)
Supplement: S1 Table — (PDF) [file pone.0174770.s001.pdf]

Maternal Age and Child Morbidity: a Danish National C

Authors: MM Hviid, CW Skovlund, L Mørch, Øjvind Lidegaard

Table S1. Summary of main diagnosis groups and ICD-10 codes

Main diagnosis group	ICD-10 codes
Bacterial infections	A00-A99
Non-bacterial infections	B00-B99
Malignant diseases	C00-C97
Benign tumors	D00-D49
Anemia	D50-D89
Endocrine disorders	E00-E90
Mental disorders	F00-F99
Cerebral diseases	G00-G99
Eye diseases	H00-H59
Ear diseases	H60-H95
Heart diseases	I00-I52
Circulatory diseases	I60-I99
Airway diseases	J00-J99
Gastro- intestinal diseases	K00-K93
Dermatological disorders	L00-L99
Rheumatic diseases	M00-M99
Kidney, urinary and genital diseases	N00-N99
Neonatal diseases	P00-P96
Congenital malformations	Q00-Q99

:ohort Study
